# Special issue on coal gasification: science and technology

**DOI:** 10.1007/s40789-020-00367-4

**Published:** 2020-09-22

**Authors:** Lu Ding, Juntao Wei, Guangsuo Yu

**Affiliations:** 1 Institute of Clean Coal Technology, East China University of Science and Technology, Shanghai, 200237 China; 2 State Key Laboratory of High-Efficiency Utilization of Coal and Green Chemical Engineering, Ningxia University, Yinchuan, 750021 Ningxia China

Coal, one of the fossil fuels which is burned for heat, contributes a quarter of the world’s primary energy and two-fifths of its electricity. According to the World Energy Model (WEM) provided by the International Energy Agency (IEA), the total primary energy demand from coal reached 3750 Mtoe in 2017, and its growth rate will decrease a lot in the future 10–20 years. Nevertheless, coal will remain as the main primary energy in the next few decades.

Coal gasification is the leading technology in achieving clean and efficient utilization of coal resources. Typical large-scale coal gasification technologies include Opposed Multi-Burner CWS gasification, GE Coal-Water Slurry (CWS) gasification, GSP pulverized coal gasification, SE pulverized coal gasification, and Shell pulverized coal gasification, etc. These coal gasification technologies can effectively improve coal utilization efficiency and reduce SO_*x*_, NO_*x*_, and CO_2_ emissions.

As the name suggests, coal gasification entails thermochemical conversion of coal into a combustible gas which is similar to natural gas. Gasification may occur in a separate processing facility or a coal mine. Although the commercialized gasifiers are widespread all over the world, continuous fundamental research is necessary to meet the technical challenges covering carbon conversion, nozzle and refractory lifetime, slagging issues and the corresponding syngas purification. Moreover, through in-depth basic research, the investment and operation cost of the actual plant will be further reduced.

This special issue publishes eleven papers related to recent advances in basic research and technological development on the processes of coal gasification. As the first step in gasification, coal pyrolysis has a close relationship with coal gasification and combustion. Due to the differences in the solid microstructure resulted from different pyrolysis processes and gas reaction kinetics, the overall gasification reaction is influenced by the intrinsic reaction rate and transportation of gases involved. Pyrolysis not only greatly affects the reactivity of the corresponding char, but also is a significant method for producing high added-value liquid and gaseous products, especially aromatic compounds, which can be separated from tar in coal pyrolysis. **The first paper** investigated effects of conventional and microwave pyrolysis on char-CO_2_ gasification. Kinetics and thermodynamic behaviors were well explored. Microwave pyrolytic char showed better thermodynamic performances with higher cold gas efficiency and CO molar concentration when compared to conventional pyrolytic char. **The second paper** evaluated the possibility of using syngas from the dry methane reforming (DMR) as a reactive gas for coal pyrolysis to improve the economics of the hydropyrolysis process by reducing the hydrogen cost. Additionally, the coal pyrolysis in pure gas atmospheres (N_2_, H_2_, CO, H_2_-CO) were conducted at 600–800 °C to investigate the mechanism of the coal pyrolysis under different atmospheres.

At present, industrial coal gasification conditions are rigorous as the processes are carried out at high temperature (> 1100 °C) and high pressure, and these will result in relatively high cost. Catalytic gasification could enhance coal reactivity and reduce temperature for the complete gasification. Therefore, it is worthwhile to develop catalytic gasification processes for lower production costs. It is well known that inorganic materials, especially AAEM, naturally existed or loaded in coal act as catalysts that improve the reactivity of gasification, alter the product distribution, and influence characteristics of char-ash-slag transition during coal catalytic gasification. **The third paper** explored effect of ash removal on structure and pyrolysis/gasification reactivity of a typical Chinese bituminous coal. The effect of ash removal by HF picking on coal structure and reactivity were investigated, and the detailed kinetic analyses were carried out. The chemical structures of coal and char were also detected, including surface morphology, functional group and carbonaceous structure. These data can provide experimental support for fundamental research concerning effects of mineral compositions on coal gasification. **The fourth paper** summarized the physico-chemical structure evolution characteristic during Yangchangwan bituminous coal (YCW) gasification in the presence of iron-based waste catalyst (IWC). The catalytic gasification reactivity of YCW was measured by thermogravimetric analyzer (TGA). IWC can catalyze YCW gasification which could provide theoretical guidance for industrial solid waste recycling. **The fifth paper** compared influence of different biomass ash additive on anthracite pyrolysis process and char gasification reactivity. It is concluded that abundant active AAEM (especially K and Na) contents of biomass ash and a lower graphitization degree of mixed chars could be responsible for the fact that the addition of corn stalk ash (CSA, high K-Na and low Si) and poplar sawdust ash (PSA, high K-Ca and high Si) improved the gasification reactivity of anthracite char.

For the conventional mining and surface gasification, the coal may experience mining, coal washing, crushing, and slurry preparation, which is time cost and energy intensive processes. The survey of energy resources was published in 2016, which estimated that the world coal reserves are approximately 890 billion tonnes, and there are another greater resources, which are not mineable in deep underground. Underground coal gasification (UCG) technology is, therefore, an option to utilize this type of coal reserve. Through this process, coal as a fuel can be extracted in a gas phase, which is known as synthesis gas or syngas. **The sixth paper** focused on the investigation of thermochemical behavior, with the measured thermal and gas products and how they are influenced by the alternation of the UCG process’s operating and boundary conditions. Comparison of findings between the results of model simulation and experimental development would will be established to provide with the necessary information for the UCG development.

Coal characters varied greatly under different coal formation conditions, and some types of coal are high in moisture and ash amount. Using them as fuel in energy-producing facilities like power plants increases coal consumption, emitting more CO_2_ and lowering power generation efficiency. Upgrading is necessary to use as a fuel for high efficiency and low emission gasification and conversion technologies. Upgrading is a process of removing moisture or ash from low-rank coals or high ash coal before using the coals to enhance facility efficiency. **The seventh paper** studied the structural evolution of Inner Mongolia lignite (IM) during hydrothermal treatment. The results showed that hydrothermal treatment is an effective method for upgrading the lignite. The side chains of the aromatic ring in lignite were altered, while the main macromolecular structure remained nearly the same. The hydrothermal treatment of IM could be divided into three temperature-dependent stages (< 493 K, 493–533 K, and > 533 K). **The eighth paper** studied the fractionation of coal through organo-separative refining for enhancing its potential for the CO_2_-gasification. A refined coal having almost zero ash contents through separative-organo refining techniques under milder ambient pressure contents could be prepared for gasification. Studies were also extended to investigate the CO_2_ gasification behavior of residual coal (RC) along with sugarcane bagasse to enhance the CO_2_ gasification reactivity.

Besides the experimental strategies, the simulations suggest that the developed numerical method is able to provide an accurate prediction on syngas formation, reaction mechanism, momentum transport, energy transport, quality transport and reaction engineering. With the help of Aspen Plus, **the ninth paper** developed a two-dimensional unsteady CFD model to simulate the coal gasification process in a fixed bed gasifier. A developed and validated two dimensional CFD model for coal gasification has been used to predict and assess the viability of the syngas generation from coal gasification employing the updraft fixed bed gasifier. The process rate model and the sub-model of gas generation are determined. The particle size variation and char burning during gasification are also taken into account. The experiments and numerical data were well compared. **The tenth paper** focused on a two-dimensional CFD simulation of a downdraft gasifier and a pilot-scale experiment for verification using wood pellet fuel. The simulation work was carried out via the ANSYS-Fluent CFD software package with in-house coding via User Defined Function (UDF). Three gasification parameters were taken into account in the simulation and validation to achieve highly accurate results; namely, fuel consumption, temperature profile, and syngas composition. **The eleventh paper** presented a numerical and experimental investigation on a fuel reactor in chemical looping combustor (CLC) system. The developed model simplified the prediction of flow patterns, particle velocities, gas velocities, and composition profiles of gas products and the distribution of heterogeneous reaction rates under the same operating conditions. The predicted and experimental results were compared according to the basis of determination coefficient (*R*^*2*^). In addition, the results showed that there was a good agreement between the predicted and experimental data.

The purpose of editing the special issue is to provide a better understanding of recent related developments, the status quo, as well as elucidating existing problems. The aforementioned will facilitate development of advanced techniques for coal gasification technology system in the future.

The editors would like to acknowledge all of the authors who contributed papers to the special issue on **Coal Gasification:Science and Technology,** especially during the special period of COVID-19. Many thanks are expressed to the academic experts who have helped the publication of this special issue by reviewing the manuscripts. Finally, we would like to thank all of our colleagues in *International Journal of Coal Science & Technology* (IJCS&T) for your always kind help and support during the proceedings of this special issue.

